# Transcriptional Responses of *Candida albicans* to Antimicrobial Peptide MAF-1A

**DOI:** 10.3389/fmicb.2017.00894

**Published:** 2017-05-17

**Authors:** Tao Wang, Jiangfan Xiu, Yingchun Zhang, Jianwei Wu, Xiaolin Ma, Yu Wang, Guo Guo, Xiaoli Shang

**Affiliations:** ^1^School of Basic Medical Sciences, Guizhou Medical UniversityGuiyang, China; ^2^Guizhou Provincial Center for Disease Control and PreventionGuiyang, China; ^3^School of Biology and Engineering, Guizhou Medical UniversityGuiyang, China

**Keywords:** *Candida albicans*, transcriptional responses, antimicrobial peptides, MAF-1A, RNA-Seq

## Abstract

*Candida albicans* is a major fungal pathogen in humans. Novel antifungal agents are urgent demanded due to the challenges of the resistance. Antimicrobial peptides (AMPs) are critical components of the innate immune system against pathogenic microorganism infection. MAF-1A is a novel cationic AMP that comes from *Musca domestica* and is effective against *C. albicans*, but the antifungal mechanism remains unclear. In this study, we performed a transcriptomics analysis in *C. albicans* using RNA-seq technique under the treatment of MAF-1A. A total of 5654 genes were identified. Among these, 1032 were differentially expressed genes (DEGs), including 575 up-regulated genes and 457 down-regulated genes. In these DEGs, genes encoding ergosterol metabolism and fatty acid biosynthesis were identified to be significantly down-regulated, while genes associated with oxidative stress response and cell wall were identified to be significantly up-regulated. Using pathway enrichment analysis, 12 significant metabolic pathways were identified, and ribosome, oxidative phosphorylation, citrate cycle were mainly involved. The results revealed that MAF-1A induces complex responses in *C. albicans*. This study provides evidence that MAF-1A may inhibit the growth through affect multi-targets in *C. albicans* cells.

## Introduction

*Candida albicans* is an opportunistic fungal pathogen of humans that can cause human mycoses, ranging from superficial mucosal and skin to invasive candidiasis, especially in immunocompromised patients (Eggimann et al., 2003; Linares et al., 2013; Guilhelmelli et al., 2016). In the past two decades, infections caused by *C. albicans* have increased significantly (Lo et al., 2015). Invasive candidiasis has an estimated mortality about 40%, even with the use of antifungal drugs (Horn et al., 2009; Lu et al., 2014). Currently, only a limited number of antifungal agents are being used to treat these infections, including azoles, polyene, and echinocandins drugs. The persistent use of these drugs have caused the emergence of drug-resistant strains. The resistance and shortage of antifungal agents have potentially limited the management of infections. Besides being pathogenic, *C. albicans* also provides an excellent eukaryotic model system to explore the antifungal mechanisms of potent drugs (Khan et al., 2015).

Recently, the therapeutic application of antimicrobial peptides (AMPs) or their analogs have received a great deal of attention (Cruz et al., 2014; Lakshmaiah Narayana and Chen, 2015; Mahlapuu et al., 2016). AMPs are relatively small (less than 100 amino acid residues), cationic, amphipathic, variable in length, sequence or structure peptides which have been isolated from animals, plants, bacteria, or fungi (Pushpanathan et al., 2013; Malmsten, 2014; Silva et al., 2014). AMPs are important components of the innate immunity system against invading pathogens. Many AMPs are effective against multi-drug resistant (MDR) microorganism and less developing resistance due to their distinguished mode of action (Seo et al., 2012). Thus, AMPs could be promising candidates for developing novel therapeutic agents against fungi.

*Musca domestica* antifungal peptide-1 (MAF-1), isolated from the instar larvae of housefly, is a kind of a novel cationic AMP with excellent antimicrobial effect (Fu et al., 2009). In previous study, full-length of MAF-1 gene was cloned, and bioinformatics analysis was used to explore structure domain and its potential physiological function (Luo et al., 2013). MAF-1A peptide was derived from the structure domain of MAF-1 and contained 26 amino acid residues (KKFKETADKLIESAKQQLESLAKEMK). MAF-1A showed its remarkable antifungal effect (Luo et al., 2013; Zhou et al., 2016), but the detailed antifungal actions of MAF-1A remain unclear.

The classic action mechanism of AMPs is cause microbial cell membrane damage. So far, most research has been focused on the use of model membrane systems such as lipid vesicles, to determine the mode of action of AMPs. Even though this knowledge is essential in our understanding of the mode of action of AMPs, it does not fully explain their interaction with microbial cell membrane or the response of microbes to the AMPs (Omardien et al., 2016). In addition, it has been proposed that some AMPs can interact with intracellular specific targets inducing cell damages, such as the inhibition of DNA, RNA, protein and cell wall synthesis (Guilhelmelli et al., 2013; van der Weerden et al., 2013; Scocchi et al., 2016; Shah et al., 2016). The analysis of microbial transcriptome may contribute to the understanding of their interaction with novel AMPs (Tavares et al., 2013). The next generation sequencing technologies for transcriptome analysis have opened the opportunity to understand a wide variety of physiological response of microorganism affected by drugs or environmental conditions treatment. RNA-Seq (deep-sequencing of cDNA) has been used successfully to identify and quantify gene expression at a genome scale level. RNA-Seq shows significant advantages such as sensitive, resolution and comprehensive, and is becoming more popular for various gene expression studies (Nagalakshmi et al., 2008; Wang et al., 2009; Nookaew et al., 2012). RNA-Seq enables genome-wide expression studies on the cellular responses and pathways of microbe affected by drug treatment via differential gene expression profiling (Heo et al., 2014; Qin et al., 2014; Le et al., 2016). This approach has already been used in the cellular responses of *C. albicans* under several different environmental conditions (Bruno et al., 2010; Cottier et al., 2015). In the present work, we investigated the differentially expressed genes and the pathways involved using high-throughput RNA-Seq technique to explore the mechanisms of MAF-1A on *C. albicans*.

## Materials and Methods

### Strain and Growth Conditions

The *C. albicans* ATCC 10231 was used throughout this study. Strain was preserved in nutrient broth supplemented with 30% glycerol stocked and stored at -80°C and streaked on Sabouraud Dextrose agar (SDA) plates (Sangon, Shanghai, China) at 37°C when used.

### Peptide Synthesis

The synthetic MAF-1A peptide is a linear peptide consisting of a 26 amino acid sequences as follows: KKFKETADKLIESAKQ QLESLAKEMK. Peptides were synthesized by Sangon Biotech Co., Ltd. (Shanghai, China). The purity of peptides was confirmed as higher than 95% using analytical reverse-phase high-performance liquid chromatography (RP-HPLC). Peptides were dissolved in sterilized de-ion water to 10 mg/mL and stored -20°C.

### MIC Determinations

Antifungal activity of MAF-1A was monitored according to Clinical and Laboratory Standards Institute (CLSI) M27-A2. Briefly, the colonies from 24 hour cultures of *C. albicans* were picked and resuspended in 0.9% sterile saline and adjusted to 0.5 Mc Farland standard (1–5 × 10^6^ CFU/mL). The yeast stock suspension was then diluted to obtain a starting inoculum of 0.5 × 10^3^ to 2.5 × 10^3^ CFU/mL. Peptides were then serially diluted in RPMI 1640 medium (Sangon, Shanghai, China) in volume of 100 μL per well, giving final concentrations ranging from 5 mg/mL to 0.1 mg/mL in sterile round bottom 96-well polypropylene microplates. Hundred microliter of standardized yeast suspension was then added to each well. After 24 h of incubation at 37°C, the minimum inhibitory concentration (MIC) was defined as the lowest drug concentration that caused 90% growth inhibition compared to drug-free growth control well by visual evaluation. Amphotericin B (Sigma, United States) and fluconazole (Sigma, United States) were used as controls. The MIC_s_ were determined in triplicate.

### Total RNA Extraction

The cells of *C. albicans* were inoculated into Sabouraud dextrose broth (SDB) medium (Sangon, Shanghai, China) and cultured at 37°C for 24 h. Before *C. albicans* were harvested for RNA extraction, the cells were treated with MAF-1A at MIC for 2 h (CA_DT). The untreated cultures were used as the control (CA_D). Total RNA was isolated with RNAiso Plus (Takara, Dalian, China) following the manufacturer’s instructions. Each RNA sample had an A260: A280 ratio between 1.8 and 2.0. RNA purity was checked using the NanoPhotometer^®^ spectrophotometer (Implen, CA, United States). RNA concentration was measured using Qubit^®^ RNA Assay Kit in Qubit^®^ 2.0 Flurometer (Life Technologies, CA, United States). RNA integrity was assessed using the RNA Nano 6000 Assay Kit of the Bioanalyzer 2100 system (Agilent Technologies, CA, United States).

### cDNA Library Construction and Illumina RNA-Seq

Libraries construction and RNA-Seq were performed by the Novogene Corporation (Beijing, China). A total amount of 3 μg RNA per sample was used as input material for the RNA sample preparations. Sequencing libraries were generated using NEBNext^®^ Ultra^TM^ RNA Library Prep Kit for Illumina^®^ (NEB, United States) following manufacturer’s recommendations and index codes were added to attribute sequences to each sample. Briefly, mRNA was purified from total RNA using poly-T oligo-attached magnetic beads. Fragmentation was carried out using divalent cations under elevated temperature in NEB Next First Strand Synthesis Reaction Buffer. First strand cDNA was synthesized using random hexamer primer and M-MuLV Reverse Transcriptase (RNase H^-^). Second strand cDNA synthesis was subsequently performed using DNA Polymerase I and RNase H. Remaining over hangs were converted into blunt ends via exonuclease/polymerase activities. After adenylation of 3′ ends of DNA fragments, NEBNext Adaptor with hairpin loop structure were ligated to prepare for hybridization. In order to select cDNA fragments of preferentially 150∼200 bp in length, the library fragments were purified with AMPure XP system (Beckman Coulter, Beverly, United States). Sequencing library quality was assessed on the Agilent Bioanalyzer 2100 system. Paired-end sequencing of cDNA was carried out with Illumina Hiseq^TM^ 2000. Raw data was filtered by removing reads with adaptor sequences, as well as low quality reads. Then, clean reads were aligned to the reference genome using TopHat v2.0.12. (Trapnell et al., 2009).

### Reads Mapping to the Reference Genome

Reference genome and gene model annotation files of *C. albicans* SC5314 were downloaded from GenBank (NW_139421). Index of the reference genome was built using Bowtie v2.2.3 and paired-end clean reads were aligned to the reference genome using TopHat v2.0.12. (Trapnell et al., 2009).

### Analysis of Differential Expressed Genes

The expression level for each gene is determined by the numbers of reads uniquely mapped to the specific gene and the total number of uniquely mapped reads in the sample. HTSeq v0.6.1 was used to count the reads numbers mapped to each gene. And then expected number of Fragments Per Kilobase of transcript sequence per Millions base pairs sequenced (FPKM) of each gene was calculated based on the length of the gene and reads count mapped to this gene (Trapnell et al., 2010).

Differential expression analysis of two conditions was performed using the DEGSeq R package (1.20.0). The *P*-values were adjusted using the Benjamini and Hochberg method. Corrected *P*-value < 0.005 and log_2_(Fold change) > 1 were set as the threshold for significantly differential expression (Wang et al., 2010).

### Enrichment Analysis of Gene Ontology (GO) and KEGG Pathways

Gene Ontology (GO) enrichment analysis of differentially expressed genes (DEGs) was implemented by the GO seq (Young et al., 2010). GO terms with corrected *P*-value < 0.05 were considered significantly enriched by differential expressed genes. KOBAS 2.0 software was used to test the statistical enrichment of differential expression genes in KEGG pathway (Mao et al., 2005). False discovery rate (FDR) of pathways was calculated. The threshold of significance of pathways was set as FDR < 0.05.

### Validation of RNA-Seq by quantitative RT-PCR (qRT-PCR)

To validate the results of RNA-Seq, eight DEGs (five down-regulated and three up-regulated) were selected for qRT-PCR. According to the SYBR Premix Ex Taq^TM^ Kit (Takara, Dalian, China) protocol, the reactions were run on an ABI7300 real-time PCR system using a 20 μL reaction system with reaction procedures of 40 cycles of 95°C for 5 s and 60°C for 30 s and 72°C for 30 s. Gene expression levels were calculated using the 2^-ΔΔCt^ method (Livak and Schmittgen, 2001) and normalized to the abundance of a house-keeping gene 18S *rRNA*. All samples were examined in triplicate. Target genes using primers listed in Supplementary Table S1.

## Results

### MIC Assay

The MIC value of the MAF-1A against *C. albicans* was 0.6 mg/mL.

### RNA Sequencing and Gene Prediction

Approximately 50,000,000 raw reads were obtained from each sample. After filtering by quality, 80.30 and 80.01% clean reads from the two groups were mapped to *C. albicans* genome. The profile of transcriptome sequence data was shown in **Table 1**. Sequence reads have been deposited in the NCBI Sequence Read Archive (SRA) under accession number PRJNA375109^1^.

**Table 1:** Profile of the transcriptome sequence data.

Sample group	CA_D	CA_DT
Raw reads	43101176	51894560
Clean reads	42085090	50808194
GC content (%)	37.60	37.09
Total mapped reads	33794586 (80.30%)	40651013 (80.01%)
Uniquely mapped reads	33095119 (78.64%)	40001046 (78.73%)
Multiple mapped reads	699467 (1.66%)	649967 (1.28%)

### Transcriptional Stress Response of *C. albicans* to MAF-1A

**FIGURE 1:**
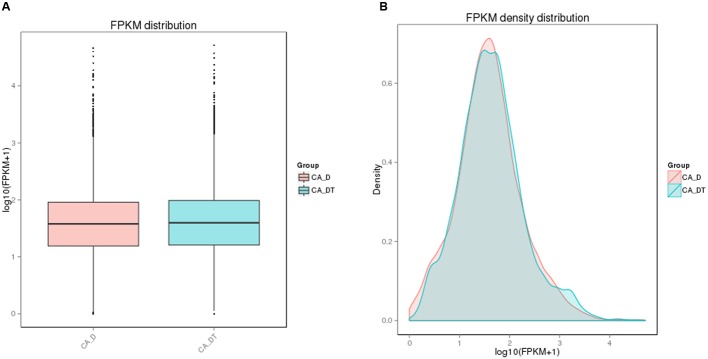
**The comparison diagram of gene expression level. (A)** FPKM (Fragments Per Kilobase of transcript sequence per Millions base pairs sequenced) distribution, **(B)** FPKM density distribution.

**FIGURE 2:**
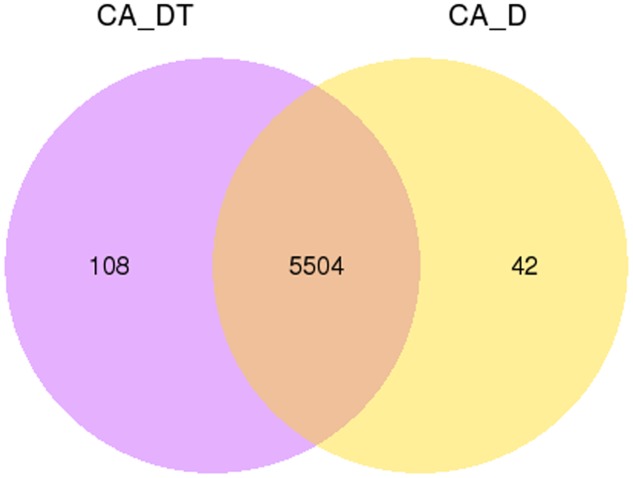
**Gene expression Venn diagram.** The number in each circle represents the total number of genes that are expressed in each sample, and the overlapping part of circles indicates that the gene is co-expressed in both samples.

**FIGURE 3:**
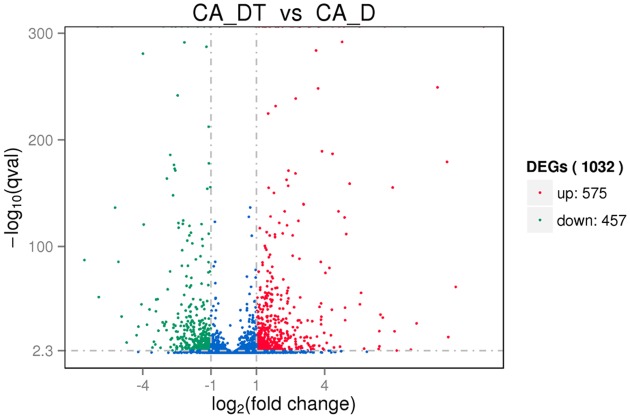
**The volcano plots of the differentially expressed genes.** Significantly differentially expressed genes were treated with red dots (up-regulated) or green dots (down-regulated), others indicated with blue dots. The abscissa represents fold change, and the ordinate represents statistical significance.

The RNA sequencing results revealed the differences in distribution and density distribution of gene expression between CA_DT and CA_D (**Figure 1**). CA_DT was detected 5612 genes expression, CA_D was detected 5546 genes expression. There are 5504 genes co-expression in these two samples, 108 genes were specificity expressed in CA_DT and 42 genes were specificity expressed in CA_D (**Figure 2**). The volcano plots of the differentially expressed genes demonstrated that 1032 genes in *C. albicans* were differentially expressed after MAF-1A treatment, with either an increase or decrease of more than twofold (the spots marked in red or green), including 575 up-regulated genes and 457 down-regulated genes (**Figure 3**).

### Verification of Differentially Expressed Genes

**FIGURE 4:**
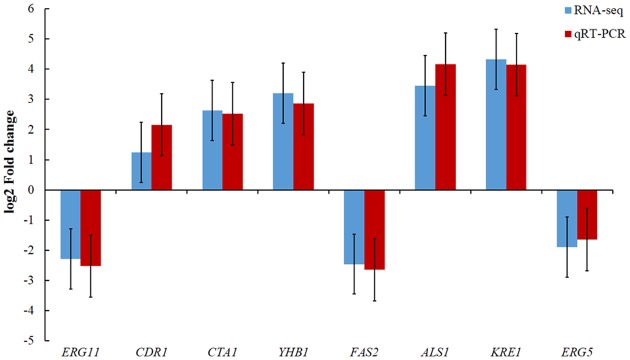
**Comparison of eight gene expression levels between RNA-Seq and qRT-PCR**.

To validate the RNA-Seq results, a total of eight genes were selected including five up-regulated and three down-regulated from DGE libraries for qRT-PCR analysis. The results indicated the expression levels have a consistent change for both RNA-Seq and qRT-PCR. Hence, the qRT-PCR results confirmed the reliability of our RNA-Seq data (**Figure 4**).

### Analysis of Differential Gene Expression

#### The Cell Wall Synthesis Related Genes

In this study, two genes involved in the biosynthesis of β-glucan (*KRE1*, *KRE6*) were up-regulated 4.32-fold, 1.31-fold, respectively (**Table 2**); one gene involved in the biosynthesis of mannoprotein (*MP65)* was up-regulated 5.73-fold (**Table 2**).

**Table 2:** The genes up-regulated or down-regulated response to MAF-1A.

Gene name	Description	log_2_Fold_change	Corrected *P*-value
**Cell wall synthesis**			
*KRE1*	β-1,6-glucan biosynthesis	4.32	1.76 × 10^-42^
*KRE6*	β-glucan synthesis-associated protein	1.31	9.06 × 10^-13^
*MP65*	Cell surface mannoprotein	5.73	2.77 × 10^-5^
**Cell membrane synthesis**			
*ERG11*	Lanosterol 14-alpha demethylase	-2.29	8.93 × 10^-30^
*ERG1*	Squalene monooxygenase	-1.30	7.30 × 10^-8^
*ERG5*	Cytochrome P450 61	-1.90	1.24 × 10^-39^
*MVD1*	Diphosphomevalonate decarboxylase	-1.01	3.05 × 10^-5^
*MET6*	5-methyltetrahydropteroyltriglutamate-homocysteine methyltransferase	-1.46	1.63 × 10^-91^
*FAS1*	Fatty acid synthase subunit beta	-1.66	1.31 × 10^-88^
*FAS2*	Fatty acid synthase subunit alpha	-2.46	2.39 × 10^-242^
**Anti oxidative stress**			
*CAT1*	Peroxisomal catalase	2.63	0.00 × 10^0^
*SOD1*	Superoxide dismutase [Cu-Zn]	1.34	1.07 × 10^-24^
*SOD4*	Cell surface superoxide dismutase [Cu-Zn] 4	1.05	3.86 × 10^-6^
*SOD5*	Cell surface Cu-only superoxide dismutase 5	1.91	5.54 × 10^-6^
*YHB1*	Flavohemoprotein	3.21	3.30 × 10^-16^
*AOX2*	Alternative oxidase 2, mitochondrial	1.77	1.22 × 10^-32^

*Corrected P-value < 0.005 were set as the threshold for significantly differential expression.*

#### The Cell Membrane Synthesis and Stability Related Genes

Several genes involved in ergosterol biosynthesis pathway were down-regulated about twofold (*ERG11, ERG1, ERG5, MET6, MVD1*). Among these genes, *ERG11*expression decreased mostly (**Table 2**).

*FAS1* and *FAS2* are fatty acid synthesis genes, which are involved in the membrane biosynthesis. After MAF-1A treatment, *FAS1* and *FAS2* were down-regulated 1.66-fold and 2.46-fold, respectively (**Table 2**).

#### Anti Oxidative Stress Genes

In this work, six genes (*CAT1, SOD1, SOD4, SOD5, YHB1, AOX2*) were identified to be up-regulated (1.03-fold to 3.21-fold) in *C. albicans* cells after MAF-1A treatment (**Table 2**).

#### Enrichment Analysis of GO and KEGG Pathways

Gene Ontology and KEGG analysis were used to classify the DEGs in the response of *C. albicans* to MAF-1A. The GO categories significantly enriched (corrected *P*-value < 0.05) among those DEGs are shown in **Tables 3**, **4**. In the DEGs, a total of 483 up-regulated and 386 down-regulated genes were classified into the following three functional categories: cellular component, molecular function and biological process, respectively. It is noteworthy that within the biological process category of up-regulated DEGs, ribosome, mitochondrion, mitochondrial part, mitochondrial inner membrane, mitochondrial envelope, mitochondrial membrane, mitochondrial membrane part were significantly enriched. The structural constituent of ribosome, oxidoreductase activity terms were enriched in the molecular function category. Oxidation-reduction process, translation terms were enriched in the cellular component category.

**Table 3:** The significant enrich Gene Ontology (GO) terms of up-regulated differentially expressed genes (DEGs).

Term_type	GO_accession	GO_term	Corrected *P*-value
Cellular component	GO:0005840	Ribosome	1.16 × 10^-6^
	GO:0044444	Cytoplasmic part	1.20 × 10^-4^
	GO:0030529	Ribonucleoprotein complex	1.03 × 10^-3^
	GO:0005739	Mitochondrion	3.70 × 10^-3^
	GO:0044429	Mitochondrial part	4.00 × 10^-3^
	GO:0005743	Mitochondrial inner membrane	8.03 × 10^-3^
	GO:0005740	Mitochondrial envelope	1.12 × 10^-2^
	GO:0031966	Mitochondrial membrane	2.30 × 10^-2^
	GO:0019866	Organelle inner membrane	2.30 × 10^-2^
	GO:0044455	Mitochondrial membrane part	2.78 × 10^-2^
	GO:0005737	Cytoplasm	3.11 × 10^-2^
Molecular function	GO:0003735	Structural constituent of ribosome	1.16 × 10^-6^
	GO:0016491	Oxidoreductase activity	6.11 × 10^-6^
	GO:0005198	Structural molecule activity	1.02 × 10^-3^
	GO:0005215	Transporter activity	5.19 × 10^-3^
	GO:0022857	Transmembrane transporter activity	9.31 × 10^-3^
Biological process	GO:0055114	Oxidation–reduction process	1.83 × 10^-4^
	GO:0006412	Translation	4.36 × 10^-3^
	GO:0055085	Transmembrane transport	4.14 × 10^-2^

*Corrected P-value < 0.05 were set as the threshold for significantly enriched.*

**Table 4:** The significant enrich GO terms of down-regulated DEGs.

Term_type	GO_accession	GO_term	Corrected *P*-value
Cellular component	GO:0005839	Proteasome core complex	1.27 × 10^-2^
	GO:0000502	Proteasome complex	2.34 × 10^-2^
Molecular function	GO:0003824	Catalytic activity	5.27 × 10^-4^
	GO:0004298	Threonine-type endopeptidase activity	3.02 × 10^-3^
	GO:0070003	Threonine-type peptidase activity	3.02 × 10^-3^
Biological process	GO:1901564	Organonitrogen compound metabolic process	7.91 × 10^-3^
	GO:0008152	Metabolic process	8.95 × 10^-3^
	GO:0044281	Small molecule metabolic process	1.76 × 10^-2^
	GO:0044710	Single-organism metabolic process	1.76 × 10^-2^

*Corrected P-value < 0.05 were set as the threshold for significantly enriched.*

By enrichment analysis, up-regulated genes were involved in 11 KEGG metabolic pathways, and down-regulated genes were involved in 1 KEGG metabolic pathway (**Table 5**). In the mapped pathways of the up-regulated genes, the abundant genes mapped onto ribosome, oxidative phosphorylation, peroxisome, carbon metabolism and citrate cycle (TCA cycle). While, the down-regulated genes were primarily related to proteasome.

**Table 5:** Differentially expressed genes were involved in significant KEGG metabolic pathways.

Regulated	Term	ID	Input number	Background number	Corrected *P*-value
Up	Ribosome	cal03010	31	140	3.34 × 10^-5^
	Oxidative phosphorylation	cal00190	26	129	6.17 × 10^-4^
	Peroxisome	cal04146	18	71	6.52 × 10^-4^
	Fatty acid degradation	cal00071	11	32	2.02 × 10^-3^
	Valine, leucine, and isoleucine degradation	cal00280	9	27	7.74 × 10^-3^
	Glyoxylate and dicarboxylate metabolism	cal00630	10	34	7.74 × 10^-3^
	Citrate cycle (TCA cycle)	cal00020	12	52	1.24 × 10^-2^
	Carbon metabolism	cal01200	26	180	1.63 × 10^-2^
	Alanine, aspartate, and glutamate metabolism	cal00250	10	47	4.02 × 10^-2^
	Fatty acid metabolism	cal01212	10	50	4.80 × 10^-2^
	Propanoate metabolism	cal00640	6	20	4.80 × 10^-2^
Down	Proteasome	cal03050	20	63	2.89 × 10^-4^

*Corrected P-value < 0.05 were set as the threshold for significantly enriched.*

## Discussion

Antimicrobial peptides are important components of natural defenses against pathogens. The mechanism of antimicrobial activity for these peptides is generally more complex. AMPs were originally proposed to act via plasma membrane permeabilization, leading to membrane rupture and rapid lysis of microbial cells. Recently, it has been proposed that AMP driven microbial death in addition to membrane disruption (Nguyen et al., 2011; Pasupuleti et al., 2012). In our preliminary work, MAF-1A was shown its remarkable activity anti-*C. albicans*. To further investigate the mechanism of actions of MAF-1A, we used RNA-Seq to study the transcriptomic profile of *C. albicans* treated with MAF-1A. The RNA-Seq results showed that the gene expression of *C. albicans* was extensively altered by MAF-1A treatment. These genes that related to cell wall, plasma membrane and anti-oxidative stress had differentially expressed comparing to control.

The fungal cell wall is essential for sustaining cell morphology and for protection against life threatening environmental conditions (Bowman and Free, 2006; Ruiz-Herrera et al., 2006). The cell wall of *C. albicans* contains three major macromolecules, including mannoproteins, β-glucan and Chitin (Klis et al., 2001). Composition of the cell wall can be quite dynamic, as it changes during cell response to environmental stresses. *C. albicans* constantly remodels the cell wall by breaking and reforming chemical bonds within and between polysaccharides to maintain integrity of the cell wall structure (Nather and Munro, 2008). *C. albicans* could change its expression of cell wall related genes to reduce the toxic effect of caspofungin (Liu et al., 2005; Nailis et al., 2010). In this work, three genes involved in the biosynthesis of cell wall were obviously up-regulated (*KRE1*, *KRE6*, *MP65*) after exposure to MAF-1A. The *KRE1*, *KRE6* encoding proteins are involved in β-glucan biosynthesis, and *MP65* encodes cell surface mannoprotein, which plays a significant role in maintaining cell wall integrity, morphogenesis and pathogenicity (Sandini et al., 2011). Therefore, the results indicated that these genes may play a role in the mitigation of cell wall damage.

The fungal plasma membrane is similar to those of other eukaryotic cells, composed of a lipid bilayer with proteins embedded within it. Sterols are major components of fungal plasma membranes. The sterol present in animal plasma membranes is cholesterol, while fungi plasma membranes contain ergosterol (Thevissen et al., 2003). This difference in sterol content is exploited in the mechanisms of antifungal agents (Spampinato and Leonardi, 2013). Itraconazole, widely used antifungal drugs, belongs to the azoles and interferes with ergosterol biosynthesis. Up-regulation of *ERG* genes were detected in *C. albicans* cells after exposure to Itraconazole *in vitro* (De Backer et al., 2001). On the contrary, *in vitro* exposure of *C. albicans* to amphotericin B (AMB) is correlated with under-expression of *ERG* genes (Liu et al., 2005). Binding to ergosterol is sufficient for antifungal activity of AMB and therefore considered the primary mode of fungicidal action (Gray et al., 2012). Besides, AMPs could kill the target cells by disrupting the integrity of fungal membranes (Bahar and Ren, 2013). In the current study, several genes (*ERG11, ERG1, ERG5, MET6, MVD1*) involved in ergosterol biosynthesis pathway were under-expression. The results indicated that MAF-1A could interfere with ergosterol biosynthesis. We speculate that down-regulation of certain ergosterol biosynthesis genes may reduce the binding of MAF-1A to ergosterol. In addition, *FAS1* and *FAS2* involved in fatty acid biosynthesis were down-regulated, indicating the composition in cell membrane was also affected by the presence of MAF-1A.

ROS, such as hydrogen peroxide and hydroxyl radicals, cause damage to proteins, lipids and nucleic acids, resulting in irreversible damage and loss of viability. A well-characterized response of eukaryotic microbes to ROS is the rapid induction of mRNAs that encode oxidative stress detoxification and repair proteins. These include catalase (*CAT1*), glutathione peroxidase (*GPX*) and superoxide dismutase (*SOD*) antioxidant-encoding genes (Dantas Ada et al., 2015). Various antifungal agents have been confirmed to cause oxidative damage to *C. albicans* (Delattin et al., 2014). For example, a number of genes involved in oxidative stress response (*YHB1, CTA1, AOX1*, *and SOD2*) were found to be up-regulated upon AMB treatment (Liu et al., 2005). In this research, such oxidative stress-responsive genes in *C. albicans* are also up-regulated following exposure to MAF-1A. These genes included *CAT1, SOD1, SOD4, SOD5, AOX2*, and *YHB1*, encodes catalase, superoxide dismutase, alternative oxidases and flavohemoprotein, respectively.

The significantly enriched GO term in the cellular component category suggest that nearly all cellular components were affected including the ribosomes, mitochondrion, cytoplasm, and organelle inner membrane. The significant enrichment of GO terms associated with molecular function indicates that the transcription levels of structural constituent of ribosome, oxidoreductase, structural molecule activity, transmembrane transporter genes were significantly changed. In addition, KEGG analysis showed that twelve metabolic pathways were affected in *C. albicans* upon MAF-1A treatment. Three metabolic pathways, including “ribosome”, “oxidative phosphorylation”, and “citrate cycle”, are the metabolic pathways of protein biosynthesis, cellular respiration and ATP generation.

**FIGURE 5:**
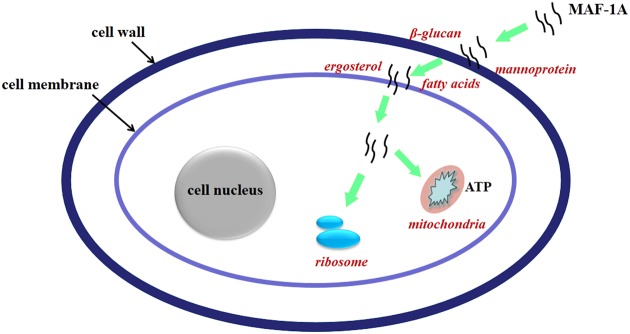
**Schematic overview of the proposed mechanisms of action of MAF-1A**.

It is widely accepted that membrane interaction is a key factor for antimicrobial activity of AMPs. Furthermore, AMPs can also act up on multiple cell targets, involving a mixed multi-hit mechanism (Guilhelmelli et al., 2013; van der Weerden et al., 2013; Silva et al., 2014; Shah et al., 2016). Considering the changes of expression pattern, it is strongly suggested that *C. albicans* inhibition caused by MAF-1A should be a result of multiple and complementary actions, involving in cell wall, plasma membrane, as well as protein synthesis and energy metabolism (**Figure 5**). It should be noted that this study has examined only on transcriptional level and further experiments are required to determine the *anti-C*. *albicans* key mechanisms.

## Conclusion

We analyzed the gene expression changes for *C. albicans* under treatment of MAF-1A using RNA-seq. The results from this study suggest that MAF-1A induces complex responses in *C. albicans* and MAF-1A has the potential to inhibit *C. albicans* growth through affect multi-targets of *C. albicans* cells. This study provides important insights into the mechanisms of action of MAF-1A and the responds of *C. albicans* under AMPs stress. However, further studies are needed to determine the key mechanisms that *C. albicans* responses to MAF-1A.

## Author Contributions

TW and JW designed the project, analyzed data and wrote the manuscript. TW, JX, YZ, and XM carried out the experiments. YW, GG, and XS reviewed the manuscript.

## Conflict of Interest Statement

The authors declare that the research was conducted in the absence of any commercial or financial relationships that could be construed as a potential conflict of interest.

## Footnotes

1 https://www.ncbi.nlm.nih.gov/bioproject/?term=PRJNA375109
