# Supplementary material for: Transcriptional Responses of Candida albicans to Antimicrobial Peptide MAF-1A
Source: Front Microbiol. 2017 May 17;8:894. doi: 10.3389/fmicb.2017.00894 (PMC5434131; doi:10.3389/fmicb.2017.00894)
Supplement: Supplementary file 1 [file Table_1.DOC]

TABLE S1. Gene-specific primers used for real-time RT-PCR assays

| Target gene | Primer forward/Primer reverse (5′-3′) | Amplicon size (bp) | Reference |
| --- | --- | --- | --- |
| *18S rRNA* | CACGACGGAGTTTCACAAGA/ | 135 | (Liu et al., 2005) |
|  | CGATGGAAGTTTGAGGCAAT |  |  |
| *ERG11* | TTTAGTTTCTCCAGGTTATGCTCA/ | 100 | (Liu et al., 2005) |
|  | ATTAGCTTTGGCAGCAGCAGTA |  |  |
| *CDR1* | ATTCTAAGATGTCGTCGCAAGATG/ | 140 | (Liu et al., 2005) |
|  | AGTTCTGGCTAAATTCTGAATGTTTTC |  |  |
| *CTA1* | ATTTCATCCACACCCAAAAGAGA/ | 79 | (Liu et al., 2005) |
|  | TTGCTAGTCAAGTAATCCCAAAACA |  |  |
| *YHB1* | AAGTTGCTCCTCCTGCTGGTAAT/ | 52 | (Liu et al., 2005) |
|  | TCCTTGTCTGTAGCTGGGTCATAGA |  |  |
| *FAS2* | ACAAATCATCAAAGGGAGTCG/ | 157 | (Higgins et al., 2012) |
|  | GCTGACCAAGTTCCAGTGAAT |  |  |
| *ALS1* | GTGCAAAGACAATCACTGGTG/ | 116 | (Sun et al., 2013) |
|  | CCAACCCAA AACAGCATTCC |  |  |
| *KRE1* | TGGGAAATTAGCCACCACCC/ | 82 | This study |
|  | ACCAGACGATGGGGTTTCAG |  |  |
| *ERG5* | GATACCGTCCACCAGTCTTGA/ | 80 | This study |
|  | TTTAGGAGCAGTGTAGGATTCAG |  |  |

**REFERENCES**

Higgins, J., Pinjon, E., Oltean, H. N., White, T. C., Kelly, S. L., Martel, C. M., et al. (2012). Triclosan antagonizes fluconazole activity against *Candida albicans*. *J*. *Dent*. *Res*. 91, 65-70.

Liu, T. T., Lee, R. E., Barker, K. S., Lee, R. E., Wei, L., Homayouni, R., et al. (2005). Genome-wide expression profiling of the response to azole, polyene, echinocandin, and pyrimidine antifungal agents in *Candida albicans*. *Antimicrob*. *Agents*. *Chemother*. 49, 2226-2236.

Sun, N., Fonzi, W., Chen, H., She, X., Zhang, L., Zhang, L., et al. (2013). Azole susceptibility and transcriptome profiling in *Candida albicans* mitochondrial electron transport chain complex I mutants. *Antimicrob*. *Agents Chemother*. 57, 532-42.
